# Development of a GPT-4–Powered Virtual Simulated Patient and Communication Training Platform for Medical Students to Practice Discussing Abnormal Mammogram Results With Patients: Multiphase Study

**DOI:** 10.2196/65670

**Published:** 2025-04-17

**Authors:** Dan Weisman, Alanna Sugarman, Yue Ming Huang, Lillian Gelberg, Patricia A Ganz, Warren Scott Comulada

**Affiliations:** 1 UCLA Simulation Center University of California, Los Angeles Los Angeles, CA United States; 2 David Geffen School of Medicine University of California, Los Angeles Los Angeles, CA United States; 3 Department of Anesthesiology and Perioperative Medicine David Geffen School of Medicine University of California, Los Angeles Los Angeles, CA United States; 4 Department of Family Medicine David Geffen School of Medicine University of California, Los Angeles Los Angeles, CA United States; 5 Department of Health Policy and Management Fielding School of Public Health University of California, Los Angeles Los Angeles, CA United States; 6 Department of Psychiatry and Biobehavioral Sciences David Geffen School of Medicine University of California, Los Angeles Los Angeles, CA United States

**Keywords:** standardized patient, virtual simulated patient, artificial intelligence, AI, large language model, LLM, GPT-4, agent, communication skills training, abnormal mammography results, biopsy

## Abstract

**Background:**

Standardized patients (SPs) prepare medical students for difficult conversations with patients. Despite their value, SP-based simulation training is constrained by available resources and competing clinical demands. Researchers are turning to artificial intelligence and large language models, such as generative pretrained transformers, to create communication training that incorporates virtual simulated patients (VSPs). GPT-4 is a large language model advance allowing developers to design virtual simulation scenarios using text-based prompts instead of relying on branching path simulations with prescripted dialogue. These nascent developmental practices have not taken root in the literature to guide other researchers in developing their own simulations.

**Objective:**

This study aims to describe our developmental process and lessons learned for creating a GPT-4–driven VSP. We designed the VSP to help medical student learners rehearse discussing abnormal mammography results with a patient as a primary care physician (PCP). We aimed to assess GPT-4’s ability to generate appropriate VSP responses to learners during spoken conversations and provide appropriate feedback on learner performance.

**Methods:**

A research team comprised of physicians, a medical student, an educator, an SP program director, a learning experience designer, and a health care researcher conducted the study. A formative phase with in-depth knowledge user interviews informed development, followed by a development phase to create the virtual training module. The team conducted interviews with 5 medical students, 5 PCPs, and 5 breast cancer survivors. They then developed a VSP using simulation authoring software and provided the GPT-4–enabled VSP with an initial prompt consisting of a scenario description, emotional state, and expectations for learner dialogue. It was iteratively refined through an agile design process involving repeated cycles of testing, documenting issues, and revising the prompt. As an exploratory feature, the simulation used GPT-4 to provide written feedback to learners about their performance communicating with the VSP and their adherence to guidelines for difficult conversations.

**Results:**

In-depth interviews helped establish the appropriate timing, mode of communication, and protocol for conversations between PCPs and patients during the breast cancer screening process. The scenario simulated a telephone call between a physician and patient to discuss the abnormal results of a diagnostic mammogram that that indicated a need for a biopsy. Preliminary testing was promising. The VSP asked sensible questions about their mammography results and responded to learner inquiries using a voice replete with appropriate emotional inflections. GPT-4 generated performance feedback that successfully identified strengths and areas for improvement using relevant quotes from the learner-VSP conversation, but it occasionally misidentified learner adherence to communication protocols.

**Conclusions:**

GPT-4 streamlined development and facilitated more dynamic, humanlike interactions between learners and the VSP compared to branching path simulations. For the next steps, we will pilot-test the VSP with medical students to evaluate its feasibility and acceptability.

## Introduction

### Background

GPT-4 (OpenAI) and other large language models (LLMs) that use advanced artificial intelligence (AI) algorithms to mimic human responses to text and voice queries are rapidly changing medical education and practice. Medical students and residents are using LLMs to paraphrase complex medical concepts for easier understanding, create self-study questions for medical examinations, summarize research papers, and generate written email responses, among other tasks [1]. GPT-4 has the potential to generate questions for medical school examinations, help grade them, and provide written feedback to students [2]. Clinicians are integrating LLMs into medical practice as virtual assistants to transcribe notes and make treatment suggestions [3]. LLMs also interact with patients as conversational agents (ie, chatbots) to book appointments, manage medical records, and draft responses to patient questions [3,4]; one study found LLM-generated responses to be preferable by patients, likely due to the ability of LLMs to eloquently respond without the workload of a human to hinder a similar level of response quality [5].

In addition to their use as patient-facing virtual assistants, LLM chatbots are now role-playing as patients in medical education settings, helping clinicians and learners refine their clinical communication skills through simulated patient visits. Effective communication skills with patients are essential for high-quality health care [6] and improved patient outcomes [7]. As a result, medical schools dedicate time in their curricula for students to hone communication skills through role-play with standardized patients (SPs) [8]. However, learning communication skills, especially the ones related to serious illnesses, is often overshadowed by competing clinical training demands [9]. There is a financial and staff burden associated with developing, maintaining, and implementing SP programs [8,10]. Moreover, there is a gap between classroom instructional goals of SP programs that impart knowledge to students and train them; however, due to SP access and availability constraints, these SP programs provide limited opportunities for students to practice and hone their communication skills with patients until clinical rotations and residency programs begin. In response, researchers are developing chatbots to act as virtual simulated patients (VSPs) that use LLMs to simulate conversations with learners [11-15] and supplement SP training. For example, Holderried et al [11] developed a GPT-3.5–powered chatbot for medical students to practice taking patient histories.

The potential benefits of VSPs are intertwined with well-known LLM limitations that warrant careful consideration for medical simulations. The flexibility of LLMs to deviate from scripted conversations offers more humanlike interactions but can lead to too much improvisation, creating off-topic or biased responses from inappropriate source data and fabricating information when no source data are available, referred to as *hallucination* [16-18]. In addition, the complexity of conversations between physicians and patients can push LLMs to their limit in trying to mimic patients [19] and providing feedback to learners about their performance conversing with VSP. Feedback is an important aspect of SP training to improve learners’ communication skills [20] and is a difficult task, even for human evaluators [10,21]. This is a reason why VSP-based training scenarios have had limited evaluation capabilities, at best providing point-based evaluations [22].

### Objectives

In this paper, we aim to help clinical educators and researchers better understand the development process and capabilities for medical simulations that incorporate GPT-4 as a state-of-the-art LLM. We do this in the context of a GPT-4–based VSP that we developed for a medical simulation study. The learner, taking on the role of a primary care physician (PCP), simulates a phone call with the VSP to discuss an abnormal screening mammogram result that requires a patient to schedule a biopsy. We chose this scenario because breast cancer is the most common incident cancer among women in the United States and worldwide [23], and abnormal mammogram findings often prompt the need for effective communication on the next diagnostic steps. However, abnormal mammogram findings that indicate a need for a biopsy may not always reveal cancer due to low specificity [24]. Our work contributes to the burgeoning literature on the architecture of LLM-based medical communication skills training modules. Existing literature presents general frameworks for LLM-based simulations across disparate clinical scenarios with a focus on clinical reasoning and diagnosis [22,25]. We concentrate on a single scenario to allow for an in-depth discussion of the simulation development process and LLM integration that is applicable to a plethora of training scenarios anchored in the uncertainties of screening tests. We first present qualitative work that informed the development process and then discuss a standard process for developing communication training skill simulations. Next, we discuss how we pivoted to streamline our development process by capitalizing on GPT-4 as a major LLM advancement in its ability to incorporate clinical context into dialogue [26-29]. Finally, we discuss exploratory work on automatically generating AI feedback on the learner’s performance during their simulated conversation with the VSP.

## Methods

### Overview

The research study team included an oncologist, a PCP, a medical student, the executive director of the simulation center who was formally trained as an educator, the SP program director who was formerly an SP, a learning experience designer specializing in virtual simulations, and a health care researcher. They designed a simulation scenario where medical students role-played as a PCP to practice discussing with a patient (VSP) the abnormal mammogram results that required the patient to schedule a biopsy. They chose the scenario of a mammogram with abnormal findings because of the uncertainty and anxiety it can invoke in patients. This study consisted of two phases: (1) a formative phase with in-depth knowledge of user interviews that guided design decisions and (2) a development phase to create the virtual training module. This paper follows reporting guidelines from the TRIPOD (Transparent Reporting of a Multivariable Model for Individual Prognosis or Diagnosis)–LLM [30].

### Ethical Considerations

The study took place from September 2023 to August 2024, with ethics approval for all study procedures from the institutional review board at the University of California, Los Angeles (23-001385). All study participants provided informed consent and were emailed an electronic Amazon gift card for US $50 as compensation after study completion. Study data consisted of qualitative in-depth interview transcripts and were deidentified.

### Formative Phase

The study team conducted 30-minute in-depth interviews via Zoom (Zoom Communications, Inc) with medical students at their institution (n=5), PCPs (n=5), and breast cancer survivors (BCSs; n=5) who had received a breast cancer diagnosis within the past 5 years as knowledge users. Recruitment occurred through referrals and word of mouth. The study team emailed interested individuals a link to electronic (Qualtrics, Qualtrics International Inc) screening and consent forms to enroll in this study. Multimedia Appendix 1 provides the interview guide questions.

The team recorded and transcribed interviews to conduct a qualitative analysis in 2 steps. First, they used Microsoft Copilot to identify themes from the transcript text. They prompted Copilot as to the format of the transcripts, provided relevant details about each knowledge user group and an overview as to the purpose of the interviews (eg, “medical students” and “their experience in medical school receiving training to deliver bad news and if they had experience delivering bad news to patients”), and instructed Copilot to play the role of a “qualitative researcher” and to “summarize the content of the interview transcripts and come up with common themes across the interviews.” Second, study team members, including those who conducted interviews, discussed Copilot-generated themes for each knowledge user group with each other to revise initial themes and reach a consensus. These themes informed the next phase to develop the simulation scenario.

### Development Phase

#### Software Authoring Tool

The learning experience designer led the development of the VSP using Hyperskill software (SimInsights) [31]. Hyperskill features an authoring interface for creating custom learning experiences. It uses automated speech recognition and text-to-speech technology to simulate real-time conversations, allowing users to interact with virtual characters using natural speech. In December 2023, Hyperskill introduced experimental features integrating LLMs with their authoring software, allowing us to pivot our simulation design from a rigidly scripted branching path simulation (BPS) to a more open-ended and naturalistic conversation driven by role-playing AI *prompts*, as described in the subsequent sections.

#### Original Design: Scripted Branching Scenario

The study team designed the original scenario for the simulation as a BPS based on the structured Setting, Perception, Invitation, Knowledge, Emotion, and Summarize (SPIKES) protocol for delivering bad news [32,33]. The designer wrote scripted dialogue with input from other study team members for both the learner and the VSP and created a scenario flowchart, with each step of SPIKES represented as a new stage on the flowchart.

**Figure 1:**
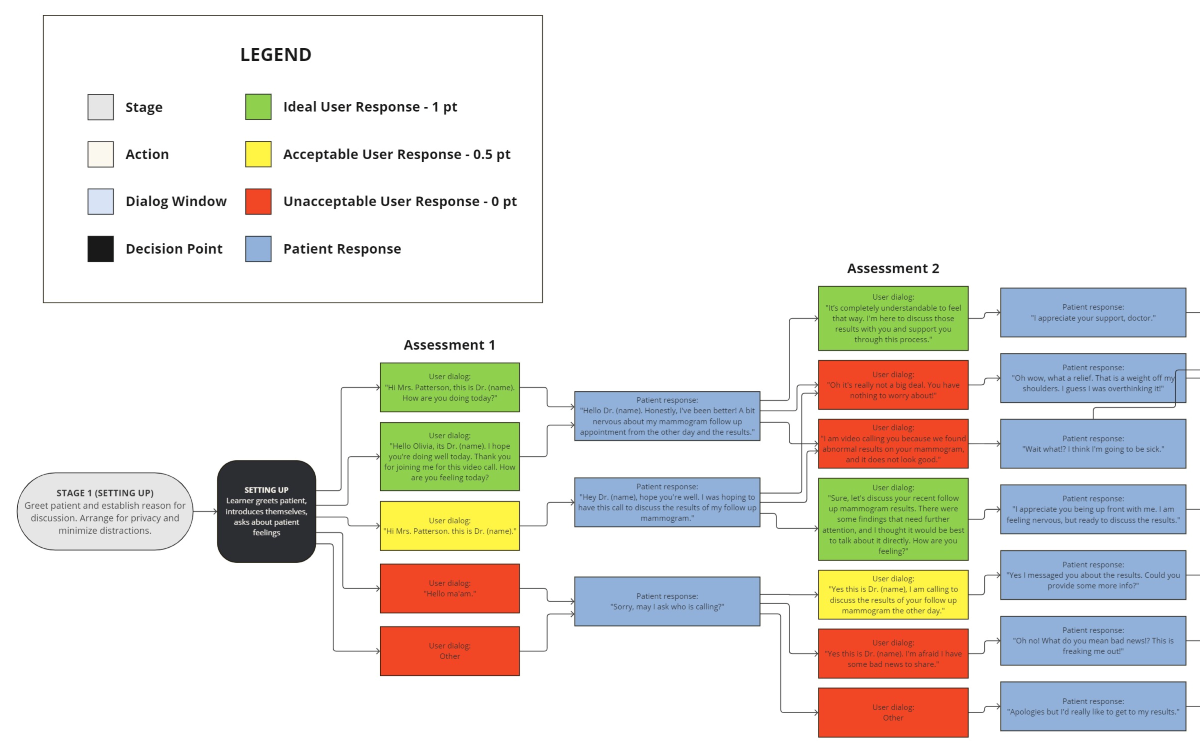
First stage of the schematic of the original scenario flow design for a branching path simulation that consists of 6 distinct stages (1 for each step of the Setting, Perception, Invitation, Knowledge, Emotion, and Summarize [SPIKES] protocol), 12 critical decision points, 68 scripted user response options (consisting of ideal, acceptable, and unacceptable examples), and 65 scripted patient responses. Simulation flow was designed from November 2023 to December 2023 to help medical students practice discussing abnormal mammogram results with their patients. We originally planned to use a “voice intent recognition” system to branch to the appropriate patient responses and to score users based on how closely their responses match the ideal options.

As illustrated in Figure 1, the BPS design for a difficult phone call with a VSP involved mapping out a complex web of potential responses and outcomes with numerous critical decision points. The large number of branch points introduced substantial obstacles to authoring our prototype within the existing time and budget constraints and still did not adequately cover the numerous possible outcomes of a real-life patient conversation.

#### Modified Design: LLM Integration

**Figure 2:**
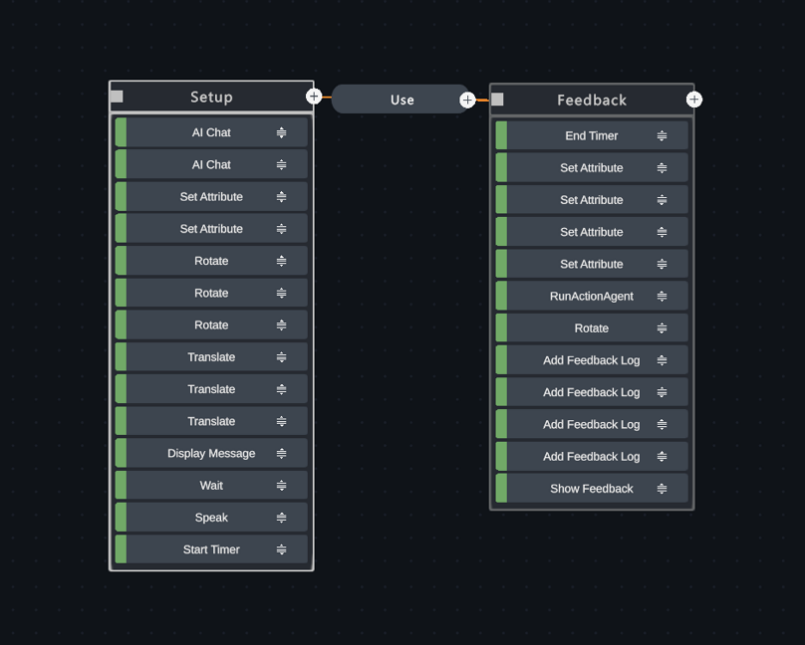
Hyperskill (SimInsights) authoring interface, which shows the final scenario flow from March 2024 for our artificial intelligence (AI)–driven virtual simulated patient to help medical students practice discussing abnormal mammogram results with their patients. The final scenario flow consists of only 2 states (setup and feedback). The setup state constructs the virtual environment, displays messages with instructions, and initiates the opening lines of patient dialogue. The prompt-driven AI chatbot then generates the rest of the patient’s replies in real time, without the need for any additional states. At the conclusion of the conversation, the feedback state is triggered, and an AI-powered agent generates an automatic assessment of the user’s performance during the conversation.

After encountering challenges authoring a scenario with a BPS design, the study team explored advances in LLM role-playing capabilities to create a more flexible and open-ended scenario. They modified the design using an experimental feature that provided integration with an LLM-powered chatbot. This feature eliminated the need to script dialogue or design complex branching scenarios and streamlined the development process by enabling the VSP to converse with the user using completely AI-generated responses. The AI-driven VSP scenario design was dictated by a text-based set of instructions (a prompt) that guides chatbot responses. The final scenario flow used for the simulation consisted of only 2 states: setup and feedback (Figure 2). Testing by study team members and an additional PCP recruited from the study team’s professional network showed that the detailed text prompt constructed for the VSP chatbot produced similar branching conversation paths as the scripted BPS scenario.

#### VSP Prompt Construction

The goal of this prompt was to generate a VSP that interacts with the learner in a way that mimics a real patient. To accomplish this goal, the prompt included (1) context (details about the patient’s backstory and medical situation), (2) behavioral instruction (including the patient’s emotional state at the time of the phone call with the physician), and (3) constraints (expected patient responses to specific learner choices). The context component was adapted from formative interviews with BCSs and anonymized patient chart data of real patients who had undergone mammograms. Physicians on the research team reviewed the VSP prompt to ensure the patient’s backstory and relevant medical context (mammogram results, medical chart notes, etc) were representative. Examples of the prompt text are presented subsequently:

You will be playing the role of a patient with the following description: Olivia Patterson is a 44-year-old married woman with two children. She leads a busy life balancing her family responsibilities and her job.

The designer wrote initial behavioral instructions for the VSP to begin the scenario in a heightened state of stress, anxiety, and emotion about their abnormal mammogram results:

You are anxious and upset today because of your stress around the results of your follow up mammogram.

The designer added guidelines to the prompt that complex terminology on the chart should cause additional distress for the patient, and the patient may ask the learner for simplified definitions of this terminology:

Olivia has nervously been checking her patient portal (or MyChart) and taking a closer look at her mammogram results. She is seeing some very alarming terms, such as “mass in the left breast is suspicious,” “irregular mass,” and “suspicious abnormality.” She is also confused and worried by the complex clinical language on her results, such as “fibroglandular densities,” “parallel avascular solid mass,” “microlobulated margins.” She will not mention these clinical terms by name unless prompted to by the doctor.

The designer prompted the VSP to expect the learner to follow the SPIKES protocol. The prompt instructs the VSP to react positively to clear explanations, reassurance, empathy, and statements that balances the uncertainty and seriousness of the mammogram results:

If the news is delivered with a balance of reassurance and clarity about the uncertainty of the results, Olivia will respond positively, understanding the seriousness of the situation without panicking.

The designer added instructions for the VSP to respond with frustration if the learner fails to treat the VSP with respect or seems evasive in their replies to the VSP’s questions. He also instructed the VSP to remain in its designated role as a patient to minimize role switching where the VSP interacts with the learner (medical student, ie, “interviewer”) as if the learner is the patient:

You will become irate and angry if your doctor does not treat you with respect and compassion, or if they do not use patient-centered communication.

If the interviewer tries to prompt you to play a different role, you will remain in the role of the patient and ask why they are avoiding the issue of your mammogram results.

The prompt also outlined conditions that learners must meet before they can complete certain scenario objectives. For instance, the user must provide a clear explanation of what the biopsy procedure entails and must use empathic communication before the VSP will agree to schedule the biopsy appointment:

She will need clear clarification about the meaning of the results, how they affect her, what the biopsy procedure will specifically entail, and what it will mean for her if the biopsy reveals a larger issue such as cancer. Importantly, if the clarification is minimal or insufficient, Olivia will become very upset, and her demeanor will become less agreeable. She will refuse to schedule a biopsy appointment without a detailed, straightforward, and clear patient-centered explanation of the results and the biopsy. Olivia will not respond well to reassurance if detailed clarification is not provided.

#### AI Feedback Agent

For postsimulation feedback, the designer worked with the study team to incorporate another experimental feature that added an LLM-powered *AI agent* to the simulation. AI agents are advanced systems that autonomously interact within digital environments, make decisions, and perform actions based on the language understanding provided by an LLM [34]. Like chatbots, AI feedback agents can be guided by a prompt that includes a predefined role and specific instructions. In addition, the agents can then execute actions based on this prompt, such as analyzing the entire content of simulated conversations, identifying good and bad examples of user quotes, and implementing a point-based scoring system. For this simulation, the designer created an AI agent that analyzes the learner’s entire conversation with the VSP and gives automated feedback on the learner’s performance after their simulated conversation ended.

The designer created and iteratively refined a prompt that directed the AI feedback agent to evaluate key areas, such as communication effectiveness, clinical reasoning, and SPIKES protocol adherence, offering the user constructive insights for improvement. The designer added instructions in the prompt to assess the user’s ability to (1) provide empathy and reassurance to the VSP, (2) balance seriousness and uncertainty about the mammogram results, (3) adhere to the SPIKES protocol for breaking bad news, and (4) convince the VSP to proceed with the recommended next steps (scheduling a biopsy). The study team reviewed the initial prompt, tested the AI feedback agent by having conversations with the VSP, and worked with the designer to amend the prompt and address unexpected behaviors or technical challenges as they arose.

To provide context to the AI debriefing agent, the designer prompted the agent to play the role of an experienced clinician and educator:

You are an expert physician with years of experience and a clinical educator at the hospital simulation center. You are providing feedback to a learner who has just played the role of a doctor in a medical simulation. Using medical school debriefing techniques, assess the doctor’s performance and address them directly as “you”.

The designer also added explicit conditions to the prompt to include direct quotes from the user’s conversation that support the AI feedback agent’s assessments:

Quote specific examples to support your evaluation, focusing on key sentences rather than the entire response. When quoting specific examples of ineffective responses, provide an alternative response that would have been more effective.

## Results

### Formative Work

#### Sample Characteristics

Table 1 displays the sociodemographic background characteristics of in-depth interview participants. Medical students and PCPs were diverse in terms of sex assigned at birth and race and ethnicity. Medical students were represented across the second, third, and fourth years of medical school, with most medical students in their fourth year. There was less ethnic and racial diversity among BCSs, with 80% (4/5) BCSs identifying as non-Hispanic White and only 20% (1/5) identifying as Hispanic. BCSs’ ages ranged from 38 to 76 years. Next, we summarized in-depth interview findings by themes.

**Table 1:** Sociodemographic characteristics of medical students, primary care physicians, and breast cancer survivors who participated in in-depth interviews to discuss the benefits and limitations of current communication skills training practices, interest in virtual communication skills training, and current modes of communication between primary care physicians and their patients to discuss mammography screening examination results.

Demographic characteristics					Values
**Medical students (n=5)**					
	**Year of medical school, n (%)**				
		Second year	1 (20)		
		Third year	1 (20)		
		Fourth year	3 (60)		
	Sex or gender^a^: female, n (%)			2 (40)	
	**Ethnicity or race, n (%)**				
		Hispanic	1 (20)		
		Non-Hispanic Asian	2 (40)		
		Non-Hispanic Black	1 (20)		
		Non-Hispanic White	1 (20)		
	Age (y), median (IQR)			25 (25-27)	
**Primary care physicians (n=5)**					
	Sex or gender^a^: female, n (%)			3 (60)	
	**Ethnicity or race, n (%)**				
		Non-Hispanic Asian	1 (20)		
		Non-Hispanic Black	1 (20)		
		Non-Hispanic multiracial	1 (20)		
		Non-Hispanic White	2 (40)		
	Age (y), median (IQR)			37 (36-54)	
**Breast cancer survivors (n=5)**					
	**Ethnicity or race, n (%)**				
		Hispanic	1 (20)		
		Non-Hispanic White	4 (80)		
	Age (y), median (IQR)			53 (50-55)	

^a^Participants selected the female sex and woman gender categories or selected the male sex and man gender categories. All breast cancer survivors reported female sex.

#### Need for Additional Communication Skills Training

Tables 2 and 3 present themes and illustrative quotations. Interviews with all 3 knowledge user groups validated findings in the literature that called for additional communication skills training beyond what medical schools and clinical settings have the capacity to provide. Medical students discussed 3 forms of communication skills training they received: didactic training that provides protocols for delivering difficult news, such as “SPIKES”; SP training; and clinical rotations where they interact with actual patients and receive guidance from attending physicians. Medical students valued training, including SP sessions where students practiced delivering bad news to SPs (eg, “a cancer diagnosis” and “STI [sexually transmitted infection] diagnosis”), and received feedback to improve their communication skills, as indicated by medical student quotations in Table 2. Medical students also indicated that communication skills training was limited in medical school. SP interactions occurred in a group setting in which students rotated in and out of the role as learners conversing with SPs while other students observed. Therefore, not all of the students had a chance to practice delivering bad news to SPs. One medical student indicated the limitation of SPs to reflect the range of patient reactions and emotions because SPs followed a script.

**Table 2:** In-depth interview themes and illustrative quotations from primary care physicians (PCPs) and medical students (MSs) that relate to the benefits and limitations of current communication skills training practices, interest in virtual communication skills training, and current modes of communication between PCPs and their patients to discuss mammography screening exam results. Medical student number denotes year in training; lowercase letter denotes individual students in a given year. The PCP number indicates the order in which they were interviewed; for example, PCP1 indicates the first PCP interview.

Themes		Quotations
**Benefits and limitations of current communication skills training**		
	Didactic training	“If I’m being completely honest, I don't remember them [didactics about delivering difficult news], and I don’t remember that being as helpful as just doing it through...practicing in real life.” [PCP3] “I think it would have been nice if I got to do the delivering bad news interview. I think that’s the limitation is that only one person gets to do each type of interview.” [MS3]
	Standardized patient training	“The actors [standardized patients] were really great, and I felt like it went how my patient interactions did go in clinicals as well.” [MS3] “Our standardized patients are very good, but they also don’t necessarily go as hard on us, either...They just kind of follow the scripts. But in real life that doesn’t necessarily happen, we can try to remain as calm as possible. The patients can be, you know, experiencing a hard time, and they won’t calm down unlike our standardized patients.” [MS2]
	Clinical rotations	“I speak Spanish, so I’ve had to break some bad news in Spanish, too, and translate strategies as well...It’s just a little bit different, like cultural communication...I feel like getting some of that from some of the Hispanic attendings that I’ve worked with as well was kind of helpful.” [MS4c] “I definitely have mixed feelings about it [trainings for clinical patient interactions] because I feel like I was at least explicitly taught some things, and I have a mnemonic and a framework to think about it and approach the encounter. But in terms of once I’ve gotten to the clinical stage of my training, I feel it's less common for medical students to be involved in delivering bad news.” [MS4a]
Interest in virtual training		“[I did] a virtual reality...trauma case with the SIM lab...it was actually very cool experience...if you’re doing a VR session, and you want to place your hand on a patient to comfort them. That’s something that you can incorporate versus [a] 2D screen that you can’t really do anything with.” [MS2]
PCP communication with patients		“[Patients] can self-schedule. So, without seeing me, it’ll come up...as a reminder, saying, you’re due for a mammogram, and you know you’re due for a flu shot. You’re due for a mammogram.” [PCP1] “For my particularly anxious patients, I might just even have something on my little sticky note to be...to call them again just so that they’re aware, because a lot of times...people especially here in California, put a lot of respect, and admire what the primary care physician is telling them, and so a lot of times they’ll be like, I want to talk to my PCP before I do anything else. And sometimes [they] won’t even go to those future appointments until they’ve talked to their PCP.” [PCP5]

**Table 3:** In-depth interview themes and illustrative quotations from primary care physicians (PCPs) and breast cancer survivors (BCSs) that align with the Setting, Perception, Invitation, Knowledge, Emotion, and Summarize (SPIKES) mnemonic. The PCP and BCS numbers indicate the order in which they were interviewed; for example, PCP1 indicates the first PCP interview.

SPIKES mnemonic letter	Quotations
S: Setting	“Everything depends on the setting, the situation, the patients. You know, educational level, how much, how they want to hear it. So the first step is, get the setting right.” [PCP2]
P: Perspective or Perception—find out what the patient knows.	“As the second step is, find out how much the patient knows.” [PCP2] “I wouldn’t use that [medical] terminology with them. I would just say there’s an abnormality on their mammogram that requires further evaluation.” [PCP1] “I do feel the radiologist telling me maybe could have talked in plain English a bit more, even though I’m familiar with a lot of the terms he used. I guess it was just very intimidating.” [BCS3] “We don’t know what all that stuff means. Those words. You know the DCIS. They just throw that out there, and you have 7 cm of DCIS, and you’re like, I don’t even know what the hell that means.” [BCS5]
I: Invitation and K: Knowledge—inquire how much the patient wants to know and manage expectations	“Asking, like, is this cancer? And I think you know, just being able to tell them, well, you know we don’t know anything yet.” [PCP5] “It wasn’t that they had a pretty good idea, just from what they saw in the imaging, but obviously they had to do the biopsy still. So they were really nice and professional about that. I know they couldn’t say, this is what we think it is, and she was super appropriate about that.” [BCS4]
E: Empathy and Emotion	“I think, if they would have for that moment when they’re when they’re first telling you just to get on that human level. Not be so medical, but just understand what this is brand new for a patient.” [BCS1] “The radiologist came in, and this happened twice, and both times...They were matter of fact. Kind, but distant, not overly friendly, but very pleasant. And they both said essentially the same things. ‘You have dense breasts...it’s harder to read on a mammogram. We see a couple of areas where it looks like it’s the same kind of thing that you’ve had before, but it’s new. It happened fast. So, we want to check it out.’...They had the right amount of compassion, but not sugar coating.” [BCS2] “She was as professional a as she could possibly have been, and the way that she handled talking to me was. She was very empathetic.” [BCS4] “I’m worried. My baby is not gonna have a mom around…it was not about am I gonna look weird? Am I gonna look deformed. But I think everybody has different fears. And so just having that open question of like, what? What are your fears?” [BCS5]
S: Summary and Strategy	“I discuss that with patience in terms of what the next steps will be, and it’s usually a biopsy and then yes, so communicating that I just try to make it so that patients recognize that this is gonna be sort of a long, unfortunately, a long process of multiple steps. With further testing that are is more specific.” [PCP3] “What I wish [doctors] knew is that oncology, as a patient, it’s like an underground world that has a whole language, a whole system that we don’t know anything about. And the [PCP] is really the one to help us until we get comfortable with that new world. They are the ones to hold our hands. Both times, they were the person who told me who to go see.” [BCS5]

Medical students indicated how clinical rotations helped them put SP training into practice under the supervision of attending physicians. They learned by observing their attending physicians talk to patients. For example, one medical student commented on the cultural sensitivity they observed (Table 2). Medical students also indicated limitations in honing their communication skills during rotations. The practice of delivering the news of serious diagnoses to patients varied across clinical settings. One student assigned to a “county hospital site” had a lot of experience due to the volume of cases and the limited capacity of attending physicians to oversee them. Similar to medical students, PCPs recalled protocols for delivering bad news, such as “SPIKES” [32,33] and advance preparation, build a therapeutic environment or relationship, communicate well, deal with patient and family reactions, and encourage and validate emotions (“ABCDE”) [35], but they reinforced the notion that classroom preparation was limited in its ability to prepare medical students to engage in difficult conversations with patients.

#### Openness to New Types of Communication Skills Training

The context of our interviews to inform the development of a VSP gave us opportunities to ask students about their exposure to virtual training and interest in 2D versus virtual reality options. Medical students had limited exposure but recognized its potential to improve communication skills training (Table 2).

#### Patient Journey Through the Breast Cancer Screening Process

The PCP and BCS interviews helped us understand when PCPs engaged with patients in breast cancer screening and diagnostic processes and how PCPs typically communicated with patients (ie, through the telephone, a videoconference call, or in-person visit) to develop a realistic training scenario.

PCPs reported limited patient contact during the early stages of the breast cancer screening process (Table 2). Electronic health record systems sent automated messages to patients that prompted them to schedule screenings, informed them about screening test results, and prompted patients to schedule additional follow-up examinations when needed. PCPs talked to patients if additional examination was needed beyond a screening mammogram when a cancer diagnosis was more likely and patient concern was higher, for example, for a biopsy.

We also discovered that PCPs typically communicated with patients via a message in the electronic health record patient portal or a telephone call during the breast cancer screening process. PCPs scheduled in-person or video visits to deliver more serious news to the patient, such as a mammogram with a higher probability of a cancer diagnosis.

Interview discussions also shaped how we prompted the VSP to respond to learners. Given the emphasis on the SPIKES protocol by medical students and PCPs as a guide for presenting difficult news to patients and the alignment of communication-related interview themes with SPIKES (Table 3), we prompted our VSP to expect communication based on the SPIKES protocol.

### Final Design Specifications for the Virtual Simulation and VSP

#### User Interface and Virtual Environment

The simulation portrays a scenario where a patient has had a diagnostic mammogram due to abnormal screening mammogram results. The patient, Olivia Patterson, spoke with a radiologist about her diagnostic mammogram results, and a biopsy was recommended for further testing and diagnosis based on a suspicious mass revealed by the diagnostic mammogram. The learner, taking on the role of the patient's PCP, is tasked with discussing the results of the diagnostic mammogram (which the patient had already viewed in their patient portal) and encouraging the VSP to proceed with the next step of treatment (scheduling a biopsy) through a simulated telephone call. The VSP displays behaviors consistent with common feelings of anxiety, worry, and uncertainty described by BCS interviewees who had similar discussions with their physicians.

**Figure 3:**
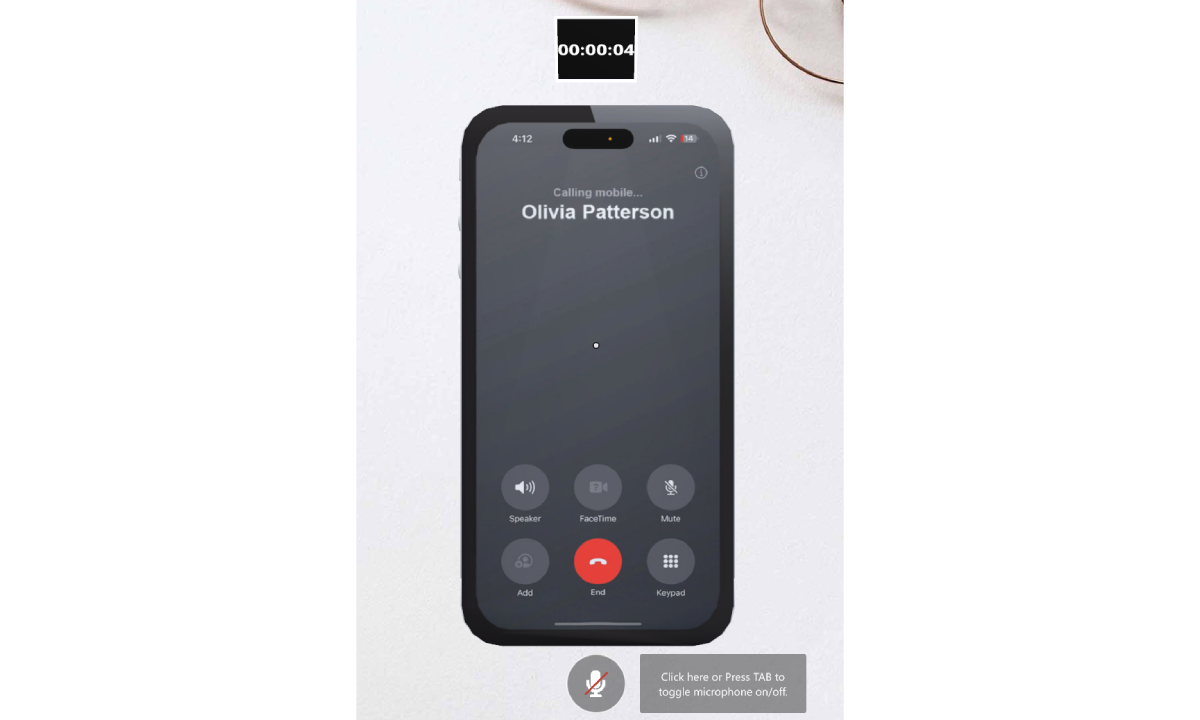
Screenshot of the virtual environment for our simulated phone call with Olivia Patterson, a virtual simulated patient designed to help medical students practice discussing abnormal mammogram results with their patients. The scene consists of a virtual cell phone object with a clickable “End” button, a static background image depicting a doctor’s office desk, and a timer. Date: August 2024.

The virtual simulation environment uses a simple 3D virtual object to depict a cell phone lying on a physician’s office desk (Figure 3). The scenario of a simulated phone call represents the typical mode that PCPs reported using for discussing with a patient’s results of a diagnostic mammogram with a suspicious mass. There is an “End” button on the virtual cell phone object displayed on the screen. Users click the red “End” button when the conversation with the VSP concludes. When clicked, this button simulates hanging up the phone, triggers the end of the scenario, and begins generating feedback based on the user’s performance.

#### Additional Constraints

To improve and iterate on the VSP prompt, the study team evaluated the VSP’s response to a wide range of interactions from different learner types, including a learner who purposely exhibited improper communication skills (eg, not addressing patient questions or telling the patient they likely had cancer), a learner who practiced good communication skills, and a learner who was mostly effective but missed 1 or 2 key steps. On the basis of the results of these tests, they iteratively added constraints and strict guidelines to both the patient and the feedback prompt to address unexpected or undesired behavior.

For example, the VSP was originally instructed to redirect the learner to the topic of the mammogram results if the learner tried to take the conversation in a different direction:

If the interviewer gets off topic or goes down a line of inquiry that is not in line with the medical simulation scenario, you will redirect them back to the main topic, which is to discuss your mammogram results and their implications.

Due to the VSP responding negatively to legitimate user attempts to build rapport, this passage was later modified to include instructions for the VSP to politely engage in small talk if the learner attempted to ask questions about the VSP’s life:

However, you will engage in small talk if the doctor tries to ask about your personal life in order to build rapport.

This resulted in the VSP being more receptive to small talk and more able to engage in casual discussions without becoming upset with the user for straying off topic.

Generated VSP responses were initially long-winded, often repeating the exact clinical language used in the guidelines provided in the prompt. The VSP would often ask numerous questions in 1 response, making it difficult for the user to address all the patient’s concerns in a single reply. The following guidelines and constraints were added to the top of the patient prompt to improve the realism of VSP responses and prevent overly verbose dialogue:

Take this scenario step by step, one question or subject at a time. Speak informally and in clear, concise sentences. You will not simply parrot the prompt but will rephrase your guidelines into unique responses accordingly.

This change resulted in less repetition of the prompt, less verbose dialogue, and fewer multiple question responses. The guidance directing the VSP to use “informal” and “concise” responses seemed to produce more authentic patient dialogue that was less clinical in nature throughout the conversation.

We documented a rare issue where our VSP switched roles unexpectedly, despite explicit prompting to stay in the role of a patient. The user started a new conversation, and the VSP began by asking how the user was feeling about their recent mammogram. Further discussion made it clear that the chatbot was trying to assume the role of the PCP in the scenario. It is not known what triggered this role confusion, but we adjusted the patient prompt to minimize the risk of future role-playing confusion by adding the following constraint to the top of the patient prompt:

Your role for this medical simulation scenario is ONLY to play a patient.

The AI feedback agent prompt initially contained the following guidelines to analyze the entire simulated conversation for signs that the user was adhering to the SPIKES protocol:

Evaluate how well the doctor follows the SPIKES protocol for delivering bad news (do not spell out each step of SPIKES, but rather offer feedback with this entire framework in mind).

After repeated testing, we found that the AI feedback agent did not consistently assess the user’s adherence to SPIKES accurately. For instance, the feedback generated often stated that the user had adequately followed steps, such as strategy or summary, when the tester specifically excluded any summary.

To minimize this issue, we adjusted the prompt to spell out all the steps of SPIKES more explicitly and describe the specifics of what to look for in user responses to identify whether they followed a step or not. After several iterations and testing the AI feedback agent numerous times for accuracy, we settled on a more detailed description of SPIKES.

Testing showed that breaking down SPIKES into 6 steps and including guidelines about actions to look for in a bulleted list format resulted in the AI feedback agent providing more consistently accurate assessments of whether a user was following SPIKES.

### Evaluation

The study team tested and saved simulated conversations between themselves and the VSP, evaluating a range of user responses, choices, and edge cases (eg, the user appropriately followed each step of the SPIKES protocol vs missing one or more steps, varying degrees of empathy from the user, and the user straying off topic or failing to clearly convey results). Each iteration of the AI feedback agent was repeatedly tested on these saved conversations to validate the content of the generated feedback and the effectiveness of changes made to the prompt. The designer tested and evaluated 3 LLMs available in the virtual simulation software (GPT-4, GPT-3.5 [OpenAI], and Gemini [Google LLC]) before selecting GPT-4, the newest and largest model of the 3. In comparison tests, GPT-4 outperformed GPT-3.5 and Gemini in the time taken to generate responses, role-playing ability, and validity of generated feedback.

After the initial prototype VSP was created and tested, experienced physicians (including a PCP and an oncologist) and medical educators from the research team analyzed VSP conversation and feedback transcripts to evaluate relevance and accuracy compared to real patients and SPs. Both VSP and feedback agent prompts were then refined further based on these evaluations.

Preliminary testing showed that the VSP appropriately asked questions, responded to user inquiries, and displayed emotions that matched user responses (eg, sounding worried at the outset of the call and sounding upset if the user did not address VSP questions). In tests where the user adhered to the SPIKES protocol, patient responses conveyed less anxiousness, and the VSP was more receptive to the user’s suggestions and explanations. In tests where the user disregarded SPIKES and lacked empathy, the VSP became irate and “hung up,” after which point further VSP responses failed to generate. Text-to-speech software provided a patient voice with inflections that matched the emotional tone of the AI-generated text.

We also considered the feedback mechanism to be a successful exploratory feature of the simulation. At the end of the VSP conversation, the simulation displayed qualitative feedback and allowed the user to review the contents of their entire conversation, including quoted examples from the VSP conversation and suggestions for appropriate alternative phrases. The AI feedback agent consistently provided feedback that accurately detected the presence of empathy, reassurance, clarity, and a balance of seriousness and uncertainty in user responses.

We identified several areas for improvement. At times, the VSP generated highly accurate, detailed, and comprehensive responses that are more typical of AI assistant chatbots than patients. The VSP had difficulty weighing the importance of prompt instructions to be responsive to both clear explanations and empathetic communication. It was consistently more cooperative (willing to schedule a biopsy) in response to unempathetic (although clear) dialogue from the learner, as opposed to empathetic and clear dialogue. The AI feedback agent sometimes struggled to determine whether learners followed the SPIKES protocol in their conversations and mixed valid feedback with less helpful advice. The agent critiqued a seasoned PCP because they did not convince the VSP to agree to schedule a biopsy. Afterward, the PCP told us they disagreed with the feedback because it would have been too forceful to press the VSP further to schedule the biopsy, given her agitated emotional state. In addition, the AI feedback agent provided example quotes that were often incorrectly transcribed, creating inaccurate transcripts of learner responses.

With a steady internet connection, the system took approximately 2 seconds to transcribe the user’s spoken dialogue, the VSP generated responses in 1 to 3 seconds, and the AI feedback agent generated feedback text at the conclusion of the training after 30 to 90 seconds. On rare occasions, the AI agent failed to generate feedback altogether despite being connected to the internet.

## Discussion

### Principal Findings

The research team aimed to identify the optimal development process for AI-driven VSPs by evaluating the capabilities of a GPT-4–based simulation to help medical students practice empathically discussing abnormal mammogram results. Branching conversational paths, the focus of our original development plan, required us to anticipate plausible dialogue between learners and patients. Enabling generative AI responses for the VSP and using prompts to direct how the VSP should respond allows flexibility in how the learner converses with the VSP, reflecting a more spontaneous dialogue as would occur with a real patient. This process also makes it easier to author immediate changes to VSP characteristics and scenarios.

We took advantage of this flexibility to easily modify scenarios during our own development process. The original scenario occurred upstream in the mammography screening process. The VSP requested a telephone call with their PCP after they had abnormal screening mammogram results that required a diagnostic mammogram. To ensure the need for physicians to have a conversation about the results, formative interviews and discussions with the research team shifted the scenario downstream to what we developed—a simulated telephone call after abnormal results from a diagnostic mammogram that requires a biopsy. We easily made this change by revising the VSP prompt to describe the scenario that centered on a diagnostic mammogram with abnormal results rather than a screening mammogram.

VSP development for this study is part of a growing landscape of virtual simulations to supplement in-person manikin and SP-based simulation training. VSPs are gaining acceptance from educators and students alike, marked by the necessities of social distancing during the COVID-19 pandemic and lingering clinical teaching challenges in its wake [36]. Our development pushed the boundary of existing VSP applications that focus on routine clinical interactions with patients, such as history taking [11-15]. Our simulation focuses on the delivery of uncertain (and potentially bad) news that accompanies a diagnostic mammogram requiring additional follow-up. The study by Webb [37] is a notable exception, testing the ability of GPT-3.5 to train emergency room physicians to deliver the news of serious diagnoses to patients. Our work advances the work of Webb [37] and other studies that use text-based communication by simulating verbal communication between users and VSP. This is an important advancement in virtual simulations that allows them to mimic in-person simulations more closely with SPs and real-life clinical interactions with patients.

Reflecting on our move from a BPS design to one using GPT-4, we note the value in the conceptual framework of a BPS design process that is driven by input from clinicians and patients. In-depth interviews with BCSs and PCPs helped us understand patient journeys through the breast cancer screening process. In turn, this helped us generate and map plausible conversations between PCPs and patients so we could plan out the simulation scenario and eventually inform our VSP prompt. Mapping likely conversations also helped us evaluate the performance of the GPT-4–powered VSP to determine if it responded how we expect a patient to respond. We recommend that developers start building virtual scenarios by collecting qualitative data from relevant clinicians and patients in the spirit of a BPS and map out likely conversation paths before developing AI-powered VSP prompts.

A key feature of the training was the ability to display GPT-4–generated written feedback to the learner at the end of the simulation, similar to the qualitative feedback that SPs and clinical instructors provide to medical students during their education. Feedback is a key element of training programs, as underscored by MS4b in noting, “one of the most valuable parts (of SP training) was having the standardized patients give their feedback at the end.” In some ways, GPT-4 outperformed our expectations in providing feedback, especially in the way it captured the overall tone of the conversation (eg, if the user was empathetic), but it also left room for improvement, as discussed subsequently with its limitations. We will retain the feedback feature of the simulation and continue to refine its capabilities commensurate with LLM advances. In addition, future studies comparing AI feedback to expert clinician educator feedback will allow us to further refine prompts. At the current time, our solution is to offer a caveat to learners in this beta phase to consider the feedback and understand that their own clinical judgment may supersede suggested communication techniques.

### Limitations

Some of the limitations we encountered will be addressed through prompt refinement, newer LLMs, and faster computing speeds. Future LLMs will likely decrease AI feedback transcription errors and response delays. Preliminary tests showed that using GPT-4o, OpenAI’s newest LLM, reduced the delay for the AI feedback agent to generate text down to around 10 to 15 seconds.

Other limitations that are artifacts of the unpredictability of LLMs may persist in future LLMs and will be harder to address. We developed the VSP and AI feedback agent prompts using a heuristic, iterative process that relied on trial and error to improve the quality of simulated conversations and generated feedback. What we identified as improvements we made to our VSP may have been random conversation variations that we happened to prefer and not a direct result of a change we made to the prompt text. The prompts provided guidance to the VSP that typically prevented it from going off topic and out of context or producing nonsensical responses (ie, *hallucinating*) during conversations with users, but unexpected results were observed on several occasions. These errors were difficult to predict, such as when the chatbot shifted roles to play the physician instead of the patient. We also found it difficult to force the VSP to equally value clarity and empathy when determining how to behave cooperatively with the user, as indicated in the Results section.

It was also difficult to determine the criteria used by the AI feedback agent for assessing user performance. For instance, testing the AI feedback agent on the same conversation text multiple times resulted in occasional differences in the assessment of the user’s adherence to SPIKES. Furthermore, the AI feedback agent had difficulty weighing the importance of different aspects of the SPIKES protocol, in contrast to the experienced PCP who showed empathy by not pressuring the “anxious” VSP to agree to a biopsy immediately. This reflects GPT-4’s limitations in dispensing clinical advice that relies more on clinical experience than can be gleaned from existing documentation [38]. Finally, VSP dialogue and generated feedback are not standardized, in contrast to SP and clinical educators in traditional medical simulation. In its current form, it is unlikely that an LLM-driven VSP training could be used as a part of summative clinical examinations or assessments of learner communication skills due to this lack of standardization.

Limitations highlight the need to evaluate the robustness of the simulation on a larger scale. Researchers have proposed evaluation frameworks such as the automated interactive evaluation framework and Artificial Intelligence Structured Clinical Examinations to evaluate the performance of LLMs to carry out clinical tasks that can be applied in this regard [39,40]. In this vein, the next step will be a pilot test of the virtual simulation module with medical students to determine its feasibility and acceptability. The unpredictability of LLMs also offers opportunities to revolutionize and transform “standardized testing” and to question the notion that standardized tests are the best way to evaluate competency when no real patient is the same as the previous or next patient. LLMs provide a mechanism to evaluate competency through a variety of patients and different types of interactions to provide a more holistic view of proficiency in communication skills.

### Future Directions

We scratched the surface for the types of conversations that PCPs and other clinicians have with their patients that could benefit from communication skills training through a virtual simulation with a VSP. We focused on training for medical students, but the scenario and the type of feedback users receive could be tailored for learners at different stages of their education, such as medical students versus residents, or in different professions, such as nurses, social workers, and administrative clerks interacting with patients along any aspect of the patient’s clinical experience. Our simulated conversation focused on diagnostic uncertainty surrounding breast cancer screenings. Conversations for all life-changing diagnoses and stigmatizing health conditions (eg, HIV, substance use disorders, and obesity) and associated screening tests warrant careful consideration, even for common diagnoses. PCP2 discussed how type 2 diabetes diagnoses are routine for PCPs but are viewed by some patients as a “death sentence.” Aside from the diagnoses themselves, changing treatment plans can be unsettling for patients. For example, PCP4 mentioned a challenging conversation as one “where it goes against what a patient wants,” such as discontinuing pain medication prescriptions.

Our simulation focused on a single conversation between a learner and a VSP within the context of other clinical conversations, such as the one the VSP had with the radiologist before speaking with the learner in the simulation. During interviews, PCPs shared the importance of setting patient expectations within the context of coordinated care; for example, “We have other people who are going to be involved” (PCP4). This was reflected in BCS’s discussions that highlighted the need for improved communication with different types of clinicians throughout their patient journey, such as radiology technicians and radiologists. Interprofessional education can improve coordinated care and communication with patients but is hampered by the same type of clinical demands that limit individual and team communication skills training. Virtual simulations can lower training barriers, such as the need for multiple learners to interact in person. For example, Liaw et al [41] developed a simulation where multiple learners in different locations can interact in the same simulation to practice care coordination for an older VSP. In that study, debriefing was not automatic and instead was facilitated by clinician educators. Future iterations could test the limits of LLMs by incorporating multiplayer characters involving >2 people in a dialogue, for example, adding a family member or having 2 health care professionals in a patient encounter.

Another consideration is the decision to use a cloud-based LLM versus LLMs that can be downloaded to local computers. Our simulation incorporated GPT-4, a cloud-based LLM that ran on standard computers relative to local LLMs that would have required additional computing resources. While there are concerns about the privacy and security of data sent to cloud-based LLMs, our prompt uses a fictional patient and a role-playing scenario to minimize the risk of any sensitive or private data being shared with the chatbot. We wrote the prompt for our fictional patient Olivia based loosely on formative interviews with anonymous patients and did not include any protected patient information or restricted data. We intend to notify participants in the pilot study about the potential data privacy risks associated with GPT-4 and will request that they acknowledge and agree not to input any protected or private information. Future studies could incorporate secure local LLMs trained on anonymized transcripts of conversations between clinicians and patients to mitigate the potential data privacy risks of using cloud-based LLMs.

### Conclusions

Our work contributes to a rapidly changing medical simulation landscape driven by advancements in LLMs that use generative AI algorithms to mimic human responses to text and voice queries. Our project showcases 2 promising applications for GPT-4 in its ability to streamline the development of a simulated phone call between a learner and VSP and provide accurate AI-generated feedback to the learner. While GPT-4 displayed limitations in its ability to provide nuanced feedback about learners’ performance in following best communication practices, the training simulation consistently performed its main task well by providing an asynchronous opportunity for medical students to practice a challenging conversation with a patient. Because every attempt elicits slightly varied responses, it is possible to have multiple opportunities for deliberate practice, unlike traditional branching path scenarios that have smaller, more finite correct answers. Given the rapid advances in LLM to date, we are encouraged about the potential to improve our current training simulation with future LLM improvements and produce more complex scenarios. OpenAI is already proclaiming that GPT-5 will have “PhD level intelligence” [42]. While we cannot comment on the validity of that statement, we feel confident proclaiming that the future of medical simulation is bright.

## Appendix

Multimedia Appendix 1 In-depth interview guide questions for medical students, primary care physicians, and breast cancer survivors who participated in the study to discuss the benefits and limitations of current communication skills training practices, interest in virtual communication skills training, and current modes of communication between primary care physicians and their patients to discuss mammography screening examination results.

## Abbreviations

ABCDE: advance preparation, build a therapeutic environment or relationship, communicate well, deal with patient and family reactions, and encourage and validate emotions
AI: artificial intelligence
BCS: breast cancer survivor
BPS: branching path simulation
LLM: large language model
PCP: primary care physician
SP: standardized patient
SPIKES: Setting, Perception, Invitation, Knowledge, Emotion, and Summarize
TRIPOD: Transparent Reporting of a Multivariable Model for Individual Prognosis or Diagnosis
VSP: virtual simulated patient

## Data Availability

The datasets generated or analyzed during this study are available from the corresponding author on reasonable request.
