# Supplementary material for: Development of a GPT-4–Powered Virtual Simulated Patient and Communication Training Platform for Medical Students to Practice Discussing Abnormal Mammogram Results With Patients: Multiphase Study
Source: JMIR Form Res. 2025 Apr 17;9:e65670. doi: 10.2196/65670 (PMC12046251; doi:10.2196/65670)
Supplement: Multimedia Appendix 1 [file formative_v9i1e65670_app1.docx]

| **Medical student in-depth interview guide questions.** |
| --- |
| **Training** |
| **Q1.** Please tell me about the communication skills training you received during your medical education to help you better communicate with patients. Please tell me about the topics you covered and training modalities (e.g., using standardized patients). PROBE: [When did you receive training, e.g,, 1^st^, 2^nd^, before clerkships, and how often, timing, frequency] |
| **Q2.** [If not already covered through Q1] Have you had any other skills training where you had to tell a patient they had an abnormal test result? |
| **Q3.** Tell me about any communication skills training you may have received during your medical education that is relevant to the situation where you would have to tell a patient that they had an abnormal mammogram. |
| **Q4.** How prepared do you feel after those trainings for clinical patient interactions like one where you would have to tell a patient that they have an abnormal mammogram? |
| **Clinical experience** |
| **Q5.** Have you had to deliver an abnormal test result to a patient? If so, tell me about the experience. |
| **Q6.** Now tell me about gaps you see in the medical school education and training you received, including communication skills training, to prepare you for clinical patient interactions like one where you would have to tell a patient that they have an abnormal mammogram. |
| **Q7.** Is there anything else you would like to add to what you’ve already told me? |

| **Primary care physician in-depth interview guide questions.** |
| --- |
| **Clinical training** |
| **Q1.** Have you received communications skills training on how to deliver bad news to patients? Do you use any systematic approaches or follow protocols to deliver bad news to patients? |
| **Mammography screening experience** |
| **Q2.** How many mammograms do you order per month, on average? |
| *Questions repeated regarding mammograms with a suspicious lesion or irregularity (asymmetry)* |
| **Q3.** Please tell me about your last experience when you had to tell a patient about a highly suspicious lesion [or irregularity] on a mammogram screening. (Probe, if needed: How long ago was that? How did the patient react?) |
| **Q4.** What did the patient express as their biggest concerns and questions after you told them that they had a suspicious lesion [or an irregularity]? |
| **Q5.** How did you communicate with the patient to address their concerns and questions? (Probe, if needed: What were the challenges you faced trying to address the patient’s concerns and answer their questions?) |
| **Q6.** Tell me how you explained the next steps to the patient for a follow-up evaluation? |
| **Q7.** After you explained the next steps for a follow-up evaluation, how did the patient react and how well do you think the patient was able to understand the next steps for a follow-up evaluation? |
| **Q8.** Based on your most recent experience and prior experiences telling patients they had an abnormal mammogram, what suggestions do you have for other physicians in how to communicate that type of news. |
| **Q9.** Are there any other aspects of abnormal mammograms you would like to tell me about? |

| **Breast cancer survivor in-depth interview guide questions.** |
| --- |
| **Q1.** How did you find out that you had an abnormal mammogram? (Probe, if needed: Method of delivery (e.g., in-person, telephone call, MyChart message) |
| **Q2.** How long ago did you find out that you had an abnormal mammogram? |
| **Q3.** Please tell me about your experience when you found out that you had an abnormal mammogram. (Prompt, if needed: What were you feeling and how did you react?) |
| **Q4.** What were your biggest concerns and questions after you found out that you had an abnormal mammogram? |
| **Q5.** Did your healthcare provider address your concerns and answer your questions after you found out you had an abnormal mammogram? |
| **Q6.** Did your healthcare provider clearly explain what the next steps were after you found out that you had an abnormal mammogram? |
| **Q7.** Based on your experience, what do you think your healthcare provider did well to address your concerns, answer your questions, and explain next steps after you found out that you had an abnormal mammogram? |
| **Q8.** Based on your experience, what do you think your healthcare provider could have done better to address your concerns, answer your questions, and explain next steps after you found out that you had an abnormal mammogram? |
| **Q9.** Is there anything else you would like to add to what you’ve already told me? |
